# A Recombinant Rabies Virus Encoding Two Copies of the Glycoprotein Gene Confers Protection in Dogs against a Virulent Challenge

**DOI:** 10.1371/journal.pone.0087105

**Published:** 2014-02-03

**Authors:** Xiaohui Liu, Youtian Yang, Zhaojin Sun, Jing Chen, Jun Ai, Can Dun, Zhen F. Fu, Xuefeng Niu, Xiaofeng Guo

**Affiliations:** 1 College of Veterinary Medicine, South China Agricultural University, Guangzhou, China; 2 State-key Laboratory of Agricultural Microbiology, College of Veterinary Medicine, Huazhong Agricultural University, Wuhan, China; 3 Department of Pathology, College of Veterinary Medicine, University of Georgia, Athens, Georgia, United States of America; Instituto Butantan, Brazil

## Abstract

The rabies virus (RABV) glycoprotein (G) is the principal antigen responsible for the induction of virus neutralizing antibodies (VNA) and is the major modality of protective immunity in animals. A recombinant RABV HEP-Flury strain was generated by reverse genetics to encode two copies of the G-gene (referred to as HEP-dG). The biological properties of HEP-dG were compared to those of the parental virus (HEP-Flury strain). The HEP-dG recombinant virus grew 100 times more efficiently in BHK-21 cell than the parental virus, yet the virulence of the dG recombinant virus in suckling mice was lower than the parental virus. The HEP-dG virus can improve the expression of G-gene mRNA and the G protein and produce more offspring viruses in cells. The amount of G protein revealed a positive relationship with immunogenicity in mice and dogs. The inactivated HEP-dG recombinant virus induced higher levels of VNA and conferred better protection against virulent RABV in mice and dogs than the inactivated parental virus and a commercial vaccine. The protective antibody persisted for at least 12 months. These data demonstrate that the HEP-dG is stable, induces a strong VNA response and confers protective immunity more effectively than the RABV HEP-Flury strain. HEP-dG could be a potential candidate in the development of novel inactivated rabies vaccines

## Introduction

Rabies remains one of the most important public health problems worldwide, causing more than 60,000 human deaths each year [1]. Rabies is caused by rabies virus (RABV), which is the prototype virus of the *Lyssavirus* genus. Rabies virus (species 1) belongs to the genus Lyssavirus in the family *Rhabdoviridae* [2]. RABV is an enveloped, non-segmented, negative-strand RNA virus with a 12 kb genome that encodes five proteins in the order of: nucleoprotein (N), phosphoprotein (P), matrix protein (M), glycoprotein (G) and the RNA-dependent RNA polymerase (L) [3]. Human rabies has been brought under control in many developed countries by routine vaccination of companion animals [4] [5]. However, routine vaccination of companion animals has not been undertaken in many developing countries. As a consequence, human rabies continues unabated [6] [7]. China is one of those countries in which companion animal immunization against rabies is not routinely performed, particularly in the rural areas [8]. The reported human rabies cases have increased to more than 3,000 cases per year during the last few years [9]. Despite the fact that the World Health Organization (WHO) does not recommend the use of live attenuated rabies vaccines for companion animals as they could potentially revert to a virulent phenotype and cause disease [10] [11], live-attenuated vaccine is being used widely in China at the present time for companion animals. It has been reported that the ERA and SAD strains used for vaccination of animals can, on rare occasions, have been linked with clinical disease in both experimental models and vaccinated wild animals. [12] [13] [14] [15] [16] [17]. Currently, the ERA strain is used in the production of live-attenuated RABV vaccine in China, contradictory to the requirement of WHO [18]. For safety reasons, WHO recommends the use of inactivated RABV vaccines prepared in cell culture with adjuvant for the vaccination of companion animals [19] [20]. However, inactivated RABV vaccines are less affordable, particularly in the developing countries [21]. It is therefore necessary to develop RABV vaccines that can be produced locally, cheap to manufacture, stable, efficacious and safe for use in companion animals in developing countries.

One way to develop an affordable RABV vaccine is to increase the virus yield and the immunogenicity. Previous reports showed that recombinant RABVs that express immune modulating molecules or apoptotic proteins have increased immunogenicity [22] [23] [24]. Since RABV G is the only viral antigen that can stimulate the production of protective VNA [25], recombinant RABV expressing more than one copy of the G-gene has also been constructed and demonstrated to stimulate higher levels of VNA than the parental virus [26] [27]. In the present study, we used the high egg passage (HEP) of RABV Flury strain to express two copies of the G-gene and showed that this recombinant RABV grows more efficiently and induces higher levels of VNA in mice and in dogs than the parental HEP-Flury virus.

## Materials and Methods

### Cell, viruses and plasmids

Baby hamster kidney (BHK-21) cells (purchased from Wuhan Institute of Biological Products) and SK-N-SH cells(SK) (purchased from cell bank in the Chinese Academy of Sciences) were grown in Dulbecco's minimal essential medium (DMEM) supplemented with 10% fetal calf serum. Recombinant RABV, rHEP-Flury strain, was derived from the HEP-Flury cDNA clone. A virus stock of the rHEP-Flury strain was prepared in BHK-21 cells as described [28]. RABV Baoding06 (BD06) strain was isolated from a rabid dog (a gift by Dr. Rongliang Hu, Laboratory of Epidemiology, Veterinary Institute, Academy of Military Medical Sciences, China). Plasmids for expression of N (pH-N), P (pH-P) , G (pH-G) , L (pH-L) and full-length genome plasmid for the HEP-Flury strain (pHEP-3.0) were kindly provided by Dr. Morimoto, National Institute of Infectious Diseases, Japan.

### Reverse transcription (RT) and polymerase chain reaction (PCR)

Genomic RNA from the virus stock was extracted using Trizol reagent (Invitrogen) according to the manufacturer's protocol. Reverse transcriptase reactions were undertaken at 42°C for 1 h with avian myeoblastosis virus RTase (TaKaRa, Japan) and oligo(dT) or rabies virus-specific primers as described below.

### Construction of full-length genome clone with double G genes

**Figure 1:**
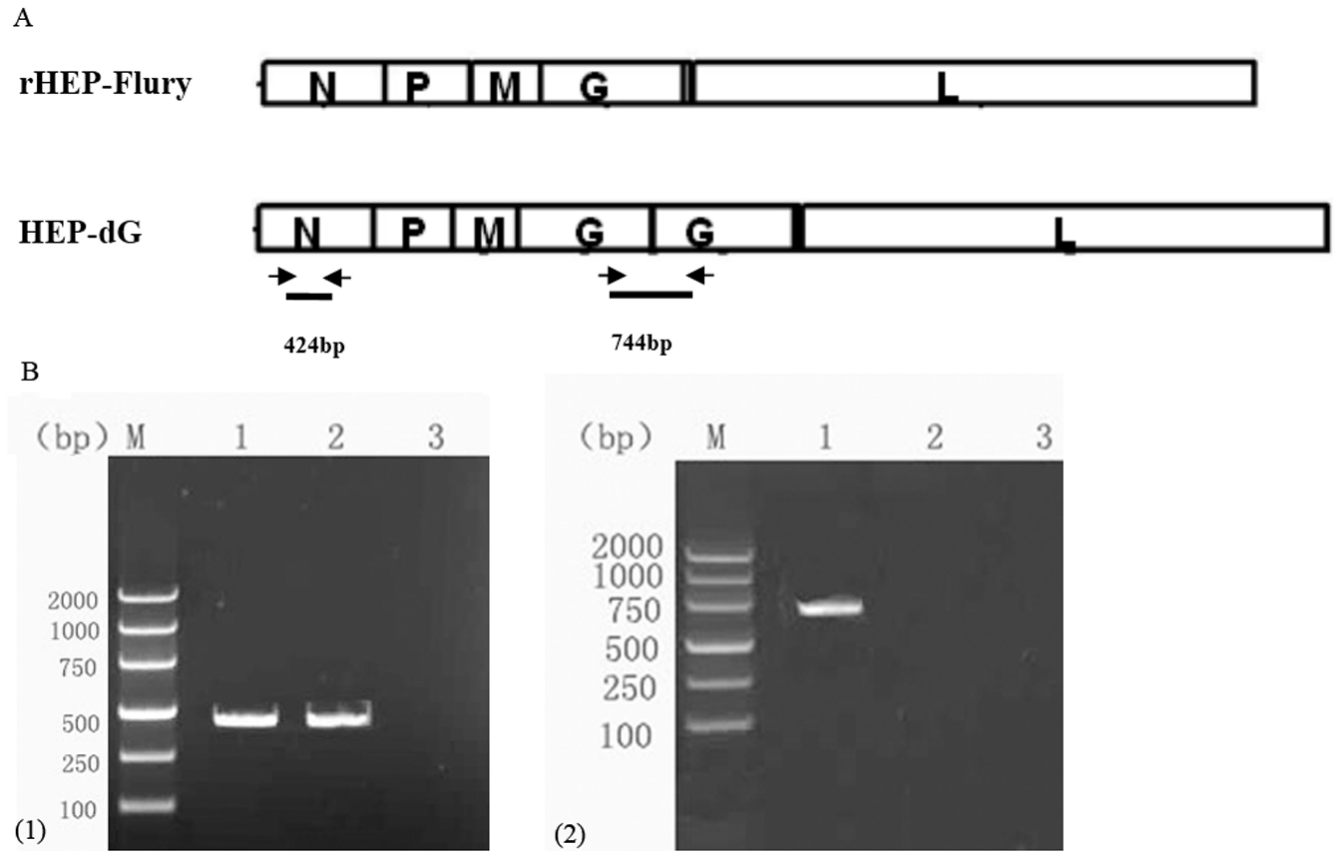
Construction of the full-length cDNA plasmid with double G gene (pHEP-dG) by RT-PCR. (A) Schematic diagram of the two virus genomes (arrows indicate the position of the two pairs of primers. (B) The RT-PCR products of the HEP-dG and the rHEP-Flury were amplified by N1/N2 primers (B1, lane 1 and 2). The RT-PCR products of the HEP-dG by DGG1/DGG2 primers after eight passages in BHK-21 cells (B2, lane 1), but no product was amplified from the cells infected with the rHEP-Flury strain (B2, lane 2). (M, molecular size marker).

To obtain the full-length genomic plasmid encoding two copies of the G genes, a 1.6-kb fragment containing rHEP-Flury G open reading frame was amplified from the template (pHEP-3.0) [29] and *BsiWI* and *PstI* restriction sites were introduced upstream and downstream of the RV G gene by using primers RVG1 (5′-ATTCGTACGATGGTTCCTCAGGTT CTT-3′; the *BsiWI* restriction site is underlined) and RVG2 (5′-ATTCTGCAGTCACAGTCTGGTCTCGCC T-3′; the *PstI* restriction site is underlined). The fragment was digested with *BsiWI* and *PstI* and cloned into pHEP-3.0 predigested with *BsiWI* and *PstI*. Fig. 1 illustrates the construction of the dG recombinant clone. The resulting plasmid was designated pHEP-dG.

### Rescue of RABV from cDNA clone

Recombinant viruses were rescued as described previously [29]. Briefly, BHK-21 cells (1×10^6^) grown in a 12-well tissue culture plate were transfected using a superfect transfection kit (Qiagen) with four helper plasmids, -N (0.625 µg), -P (0.3125 µg), -L (0.125 µg), -G (0.1875 µg) and the full-length cDNA clone pHEP-dG (2.5 µg). At day 6 after transfection, supernatants were transferred to a 96-well plate and incubated for another 6 days. Cells were examined by immunofluorescence staining with FITC-labeled RV N-specific antibody (Fujirabio Inc. Malvern, PA). The culture fluid was collected as virus stock and stored at −80°C until use.

### Confirmation of the rescued virus by RT-PCR and sequencing

To confirm if the rescued RABV was derived from pHEP-dG, RT-PCR was preformed with two pairs of primers. The primer pair N1 (sense) (5′-AGTCTCTATAGGTTGAGC-3′) and N2 (antisense) (5′-GATGAAATAAGAGTGAGG-3′), corresponding to the positions from nucleotide 506 to 524 and from 930 to 948 of the N gene were used to amplify RABV N gene. Another primer pair DGG1 (sense) (5′-AAAGGGTGTTTGAGAGTTGG-3′) corresponding to the positions from nucleotide 1079 to 1099 (based on the first G gene sequence of the HEP-dG genome) and DGG2 (antisense) (5′-ACAGGTTGGTACATCCTTCGTCC-3′) corresponding to the positions from nucleotide 149 to 172 (based on the second G gene sequence of the HEP-dG genome) was used to amplify the double G gene of HEP-dG virus. Sequencing of the amplified cDNA fragment was carried out by using TaKaRa reagents.

### Titration of virus

Viral titers were determined by direct fluorescent antibody assay in BHK-21 cells. BHK-21 cells in 96-well plate were inoculated with serial 10-fold dilution of virus and incubated at 37°C for 4 days. Cells were fixed with 80% acetone for 30 min and stained with FITC-labeled anti-rabies mAb (Fujirabio). Antigen-positive foci were counted under a fluorescent microscope (OLYMPUS) and calculated as focus forming unit (FFU) per milliliter.

### Virus growth curve

Monolayer cultures of 5×10^6^ BHK-21 cells were infected with individual virus at a multiplicity of infection (MOI) of 5. Then the cultures were incubated at 37°C. Supernatants were harvested at 1, 2, 3, 4 and 5 days post-inoculation (p.i.), and virus titers determined by the fluorescent-focus assay as described above.

### mRNA expression of G-gene

Once BHK-21 cells had grown to cover 90% of the culture flask, at an input multiplicity of infection (MOI) of 1, the seed of the rabies rHEP-Flury and HEP-dG were inoculated. After 6, 12, 48 and 96 h, cells were collected and used for Q-PCR.

### Western blot analysis

Western blotting was performed as described previously [30]. Briefly, BHK-21 cells grown in tissue culture flasks were infected with HEP-dG and rHEP-Flury at an MOI of 1. After 24, 48, 72 and 96 h, cells were collected and lysed with lysis buffer (10 mM Tris–HCl, pH 7.4, 150 mM NaCl, 1% Triton X-100 and 0.5% sodium deoxycholate). The lysates were mixed with an equal volume of 2× loading buffer (100 mM Tris-HCl [pH 6.8], 200 mM dithiothreitol, 2% SDS, 0.1% bromophenol blue and 10% glycerol), and the proteins were resolved by sodium dodecyl sulfate–12% polyacrylamide gel electrophoresis and transferred onto a polyvinylidene difluoride (PVDF) membrane. Mouse anti-rabies virus G monoclonal antibody (Chemicon, USA), Mouse anti-rabies virus N and P monoclonal antibody (Zhejiang Tongdian Biotechnology Co. LTd, P.R. China), β-actin mAB reagent (bioworld) and horseradish peroxidase (HRP)-conjugated goat anti-mouse IgG (Sigma, USA) were used respectively as the primary and secondary antibodies at the dilutions recommended by the manufacturers. A molecular imager (CN-3000 Infinity, France) was used to detect the protein bands.

### Virus focus

BHK-21 cells were maintained in Dulbecco's modified Eagle's medium (DMEM) supplemented with 0.1 mg/ml Penicillin-Streptomycin and 10% fetal bovine serum (FBS). When the BHK-21 cells had grown to cover 90% of the culture flask, they were digested by trypsin 0.25% EDTA and then, at an MOI of 1, they were inoculated with the seed of the rabies rHEP-Flury and HEP-dG. Virus infected cells were grown in triplicate with non-infected cells acting as the negative control. At 6, 12 and 36 h post-infection (hpi), cells were fixed in 80% acetone and stained with FITC-labeled RABV N protein-specific antibody (Centocor, Inc), and examined under a fluorescence microscope.

### Assay of apoptosis

For Annexin V/PI assays, cells were stained with Annexin V-FITC and PI, and evaluated for apoptosis by flow cytometry according to the manufacturer's protocol (Annexin V-FITC Apoptosis Detection Kit, vazyme). Briefly, 1×10^5^ BHK-21 cells and SK-N-SH cells were washed twice with PBS (pH 7.4, 137 mM NaCl, 2.7 mM KCl, 10 mM Na2HPO4 and 2 mM KH2PO4), and stained with 5 µL of Annexin V-FITC and 5 µL of PI, respectively, for 10 min at room temperature in the dark. Finally, 500 µl of binding buffer was added to each tube and the apoptosis was detected by flow cytometry (BD FACS Aria, USA) within 1 hr.

### Pathogenicity of RABV in suckling and adult mice

Virulence of the HEP-dG and rHEP-Flury viruses was determined by inoculating 3-day-old ddY mice (purchased from Southern Medical University, Guangzhou, China, ID0062265). Groups of suckling mice were inoculated intracerebrally with 20 µL of serial 10-fold dilutions of each virus. The fifty percent lethal dose (LD_50_) of each virus was calculated by the methods of Reed and Mueeh [31]. The pathogenicity in adult mice was determined in mice at various ages infected intracerebrally with 30 µL of HEP-dG or rHEP-Flury virus containing 5×10^4^ focus-forming units (FFU). Groups of ten mice were used for each virus.

### Immunization of mice with inactivated RABV

Six-week-old female ddY mice were immunized with inactivated viruses. Stock viruses were inactivated with 0.025% β-propiolactone. Groups of ten adult mice were inoculated with 0.5 ml of inactivated viruses (equivalent to 10^7^FFU/ml). Mice were bled from the tail vein every week for four weeks, and the serum was used to determine VNA titers. Immunized mice were challenged intracerebrally with a lethal dose of RABV Pasteur virus (PV) strain (China institute of veterinary drug control).

Another thirty adult female ddY mice were divided into four groups(grouped A, B, C,D). Each mouse in group A,B,C was immunized with 0.5 ml of inactivated viruses mixed with 10%V/V Montanide PET GEL adjuvant, and mice in group D were injected with 0.5 ml DMEM. The inactivated stock viruses in group A, B and C were equivalent to 10^6^FFU/ml, 10^7^FFU/ml, and 10^8^FFU/ml. At day14 post-immunization, mice were bled and the VNA titers were detected.

### Immunization and challenge of dogs

Adult dogs (3 months of age, purchased from Gaoyao Kanda Laboratory Animal S and T Co,, Ltd) were divided into 3 groups. Dogs in group A were inoculated subcutaneously with 1 ml of inactivated HEP-dG (equivalent to 10^7^FFU/ml) mixed with 10%V/V Montanide PET GEL adjuvant (provided by SEPPIC China) and dogs in B with 1 ml of inactivated rHEP-Flury and dogs in C were sham-immunized with 1 ml of medium. After immunization, each dog in groups A, B and C (five dogs/each) was bled intravenously every week for four weeks and the serum was used to determine VNA titers.

Another 65 adult dogs (3 months of age) were divided into two groups (grouped D and E). Dogs in group D were inoculated subcutaneously with 1 ml of inactivated HEP-dG (equivalent to 10^7^FFU/ml) and dogs in group E were sham-immunized with 1 ml of medium. On the 21th day of post-immunization, 40 dogs in groups D and 25 dogs in group E were challenged with 1 ml of RABV BD06 strain (10^5^LD_50_/ml) in the masseter muscle (Clinical trials of veterinary biological products permitted by Chinese Ministry of Agriculture, ID 201035)

In an another experiment fifteen adult beagle dogs were grouped. Dogs in group A, B and C were injected respectively with 0.25 ml, 0.5 ml and 1.0 ml inactivated HEP-dG(10^8^ FFU/mL) mixed with 10%V/V Montanide PET GEL adjuvant and dogs in group D was treated with 0.5 ml DMEM. At day 14 post-immunization the serum were collected from vein and VNA was determined.

### Comparison of antibody level

Sixteen adult beagle dogs were divided into two groups (HEP-dG and G52 with eight dogs in each group). Dogs in group HEP-dG were inoculated subcutaneously with 1 ml of inactivated HEP-dG (equivalent to 10^7^FFU/ml) mixed with 10%V/V Montanide PET GEL adjuvant (provided by SEPPIC China) and dogs in group G52 with 1 ml of inactivated commercial vaccine G52 strain (a licensed vaccine in China, produced in Merial SAS. France, batch number L374912) After immunization, each dog in group HEP-dG and G52 was bled intravenously at days 14, 21 60, 120, 180, 240, 270 and the serum was used to determine VNA titers.

### Persistence of antibodies after immunization

To determine the persistence of anti-RABV antibodies, five 3-month-old dogs were vaccinated subcutaneously with 1 ml inactivated virus vaccine (10^7^FFU/ml) mixed with 10%V/V Montanide PET GEL adjuvant (SEPPIC China). Serum samples were collected from each dog at the days 0, 14, 21, 60, 90, 120, 150, 180, 210, 240, 270, 300, 330 and 360 post-immunization and VNA titers determined (Clinical trials of veterinary biological products permitted by Chinese Ministry of Agriculture, ID 201035).

## Results

### Rescue of the recombinant RABV expressing double G genes

To recover the recombinant RABV, the full-length genomic cDNA clone pHEP-dG and helper plasmids pH-N, pH-P, pH-L and pH-G were co-transfected into BHK-21 cells and incubated for 2 days at 37°C with 5%CO_2_ and then at 34°C for 4 days. After freeze-thawing the cells three times, the supernatant and fresh BHK-21 cells were mixed, plated into a 96-well plate, and incubated for 2 days at 37°C, and then 4 days at 34°C. The cells were fixed with 80% acetone, stained with FITC-labeled anti-N antibody, and observed using a fluorescence microscope. Positive fluorescent spots were observed in the cells, indicating that recombinant double G RABV was rescued. It was designated as HEP-dG. To confirm that the recovered RABV was the recombinant RABV encoding double G genes, the viruses were passaged ten generations in BHK-21 cells and were confirmed by RT-PCR with one pair of primers (N1/N2) to amplify the conserved N-gene sequences and another pair (DGG1/DGG2) to amplify the G-G gene non-coding region. A fragment of 424 base pair (bp) were amplified by the N1/N2 primers when RNA was extracted from either parental rHEP-Flury or the recombinant virus HEP-dG (Fig. 1.B1). When primers of DGG1/DGG2 were used, a fragment of 744 bp was amplified only from HEP-dG, (Fig. 1.B2, lane 1), but not from rHEP-Flury (Fig. 1.B2, lane 2), which indicated that the dG recombinant virus contains the double G-genes.

### Characterization of HEP-dG virus in vitro

**Figure 2:**
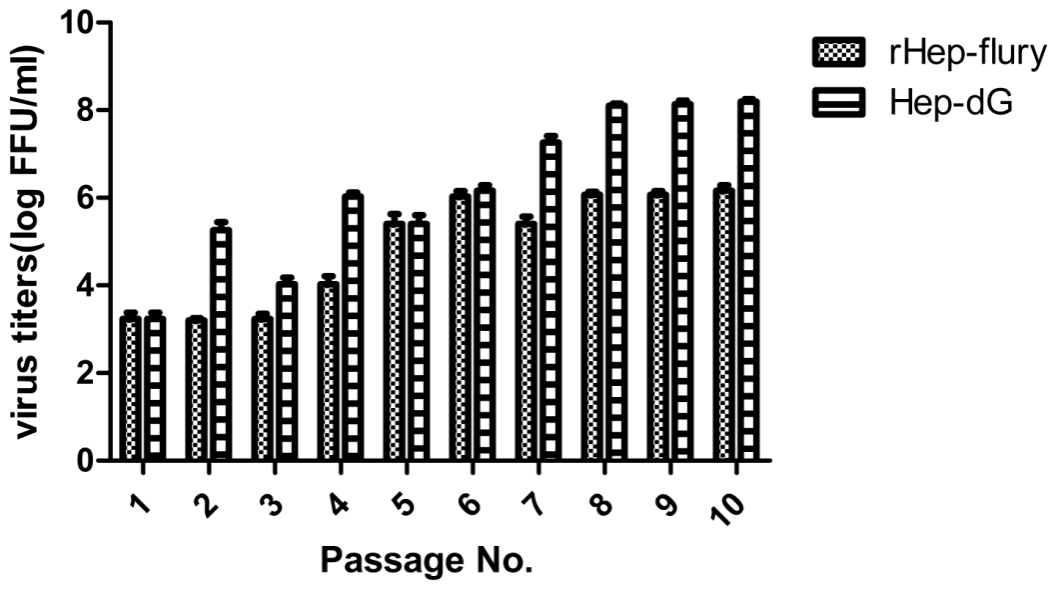
Virus titers of HEP-dG and rHEP after serial passagings in BHK-21 cells. BHK-21 cells were inoculated with HEP-dG or rHEP-Flury at an MOI of 5 and the culture fluid were harvested at 4 days post inoculation for virus titration.

To characterize HEP-dG virus in vitro, HEP-dG and the parent virus were passaged serially in BHK-21 cells and the virus titers determined at each passage. It was found that the titer of both viruses increased gradually and by the 8^th^ passage, the titer of HEP-dG reached 10^8.2^FFU/ml, but that of the parental virus peaked at the 8^th^ passage and remained at 10^6.2^FFU/ml up to the tenth passage (Fig. 2), indicating that the dG virus grows better than the parent virus in BHK cells. At the eighth passages, viral RNA was extracted from dG-infected cells and subjected to RT-PCR and direct sequencing, it was confirmed that the recombinant virus contained double G-genes, indicating that the HEP-dG recombinant virus is stable.

**Figure 3:**
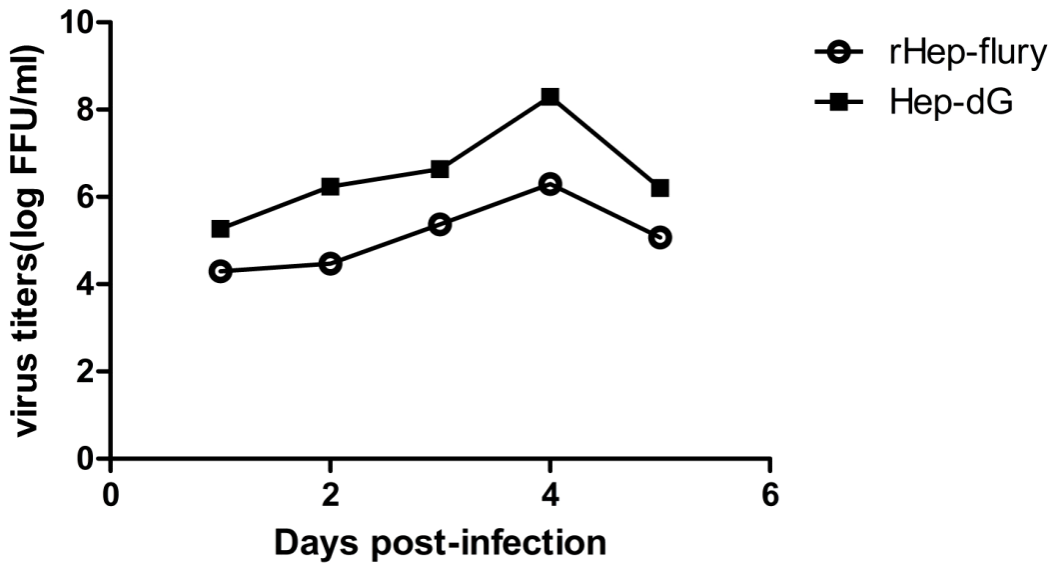
Growth kinetics of HEP-dG and rHEP-Flury in BHK-21 cells. Cells were infected with HEP-dG (▪) and rHEP-Flury (**○**) at a MOI of 5. The cultures were incubated at 37°C. Supernatants were harvested at 1, 2, 3, 4 and 5 days post inoculation, and virus titers examined by the fluorescent-focus assay.

To examine the growth properties of the recombinant RABV, the growth kinetics of the parental rHEP-Flury strain and HEP-dG strain were determined in BHK-21 cells. As shown in Fig. 3, peak virus titers were detected at 4 days after infection for both viruses. At this stage, the titer of the recombinant virus HEP-dG reached 8.0×10^8.2^ FFU/ml, which was 2 logs higher than that of the parental rHEP-Flury. At days 1,2,3 and 4, the titer of HEP-dG virus in BHK-21 cells was significantly higher than rHEP-Flury virus (*p*<0.05, independent-samples T Test using the Statistical Package for Social Sciences (SPSS®), version 17.0.)

**Figure 4:**
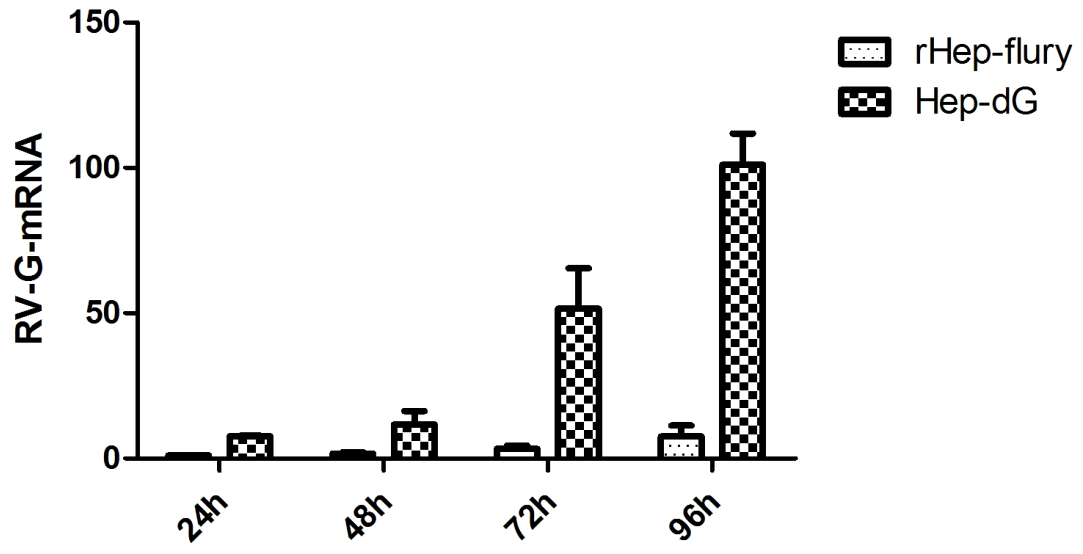
Q-PCR analysis of mRNA expression of G-gene. BHK cells was infected with virus HEP-dG and rHEP-Flury. The cells were collected and the numbers of G-gene mRNA were detected post-infection 24, 48,72 and 96 h.

To understand the difference in the G gene mRNA expression between the rabies viruses, rHEP-Flury and HEP-dG, the total mRNA in BHK-21 cells infected with rHEP-Flury and HEP-dG was extracted. Q-PCR was used to analyze the mRNA of the G gene and GADPH. At 6, 12, 48 and 96 h post-infection, the amount of mRNA of the G gene in the cells inoculated with the HEP-dG was higher than rHEP-Flury. Expressly at 96 h, the difference was extremely significant (p<0.01, independent-samples T Test using the Statistical Package for Social Sciences (SPSS®), version 17.0.) (Fig. 4).

**Figure 5:**
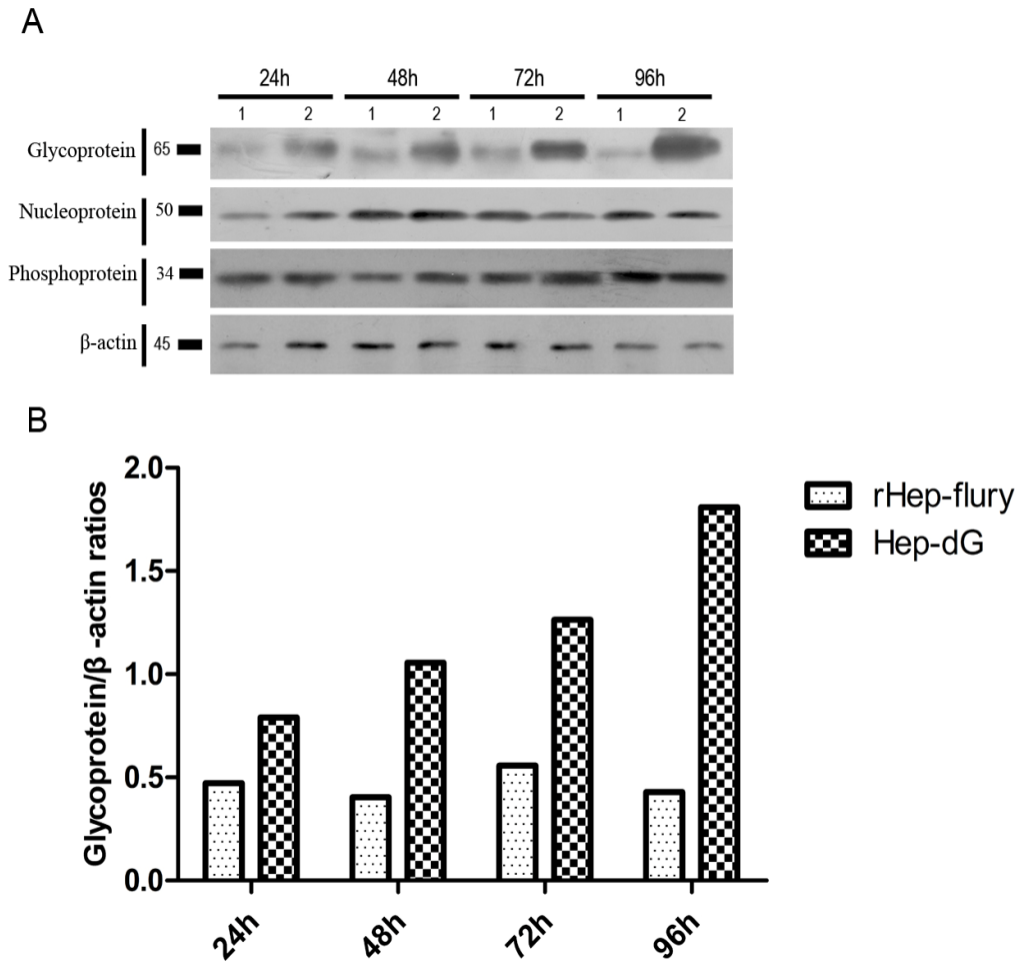
Western blot analysis of RV proteins. BHK-21 cells were infected with HEP-dG and rHEP-Flury at an MOI of 1. After incubation for 24, 48, 72, 96 h at 37°C, the cells were lysed and proteins separated by SDS-PAGE. The G, N and P proteins of HEP-dG(lane 2) and rHEP-Flury(lane 1) and β-actin were detected by mouse anti-rabies virus G, N and P monoclonal antibody and β-actin mAB reagent (A). The relative amounts of G protein were determined by a molecular imager(B).

HEP-dG and rHEP-Flury were used to infect BHK-21 cells to determine if an additional G gene improved the G protein expression and affected the N and P protein expression. After incubation at 37°C for 24, 48, 72 and 96 h, the cells were collected and the lysates were subjected to Western blot analysis with mouse anti-G, N and P monoclonal antibody and β-actin mAB reagent. The G protein was expressed at a higher level in the HEP-dG than in the rHEP-Flury, but the N and P protein were not different in the two virus strains, as shown in Fig. 5A. Grayscale analysis of the protein bands on the membrane revealed that the HEP-dG glycoprotein level in the BHK-21 cells was 1.85, 2.08, 1.67 and 3.74 fold higher than that in the rHEP-Flury at 24, 48, 72 and 96 hpi, respectively (Fig. 5B).

**Figure 6:**
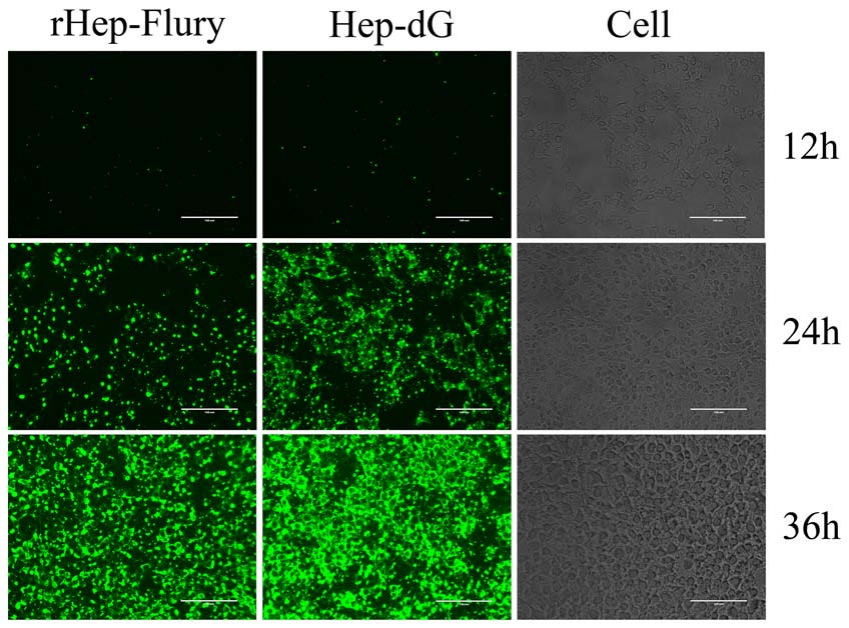
Foci of virus infection in BHK cells. The BHK cells were inoculated with virus HEP-dG and rHEP-Flury and stained with FITC-labeled anti-rabies mAb at post-infection 12, 24 and 36 h. Antigen-positive foci were observed under a fluorescent microscope.

Immunoflorescence assay analysis was performed on the infected BHK-21 cells to enable an understanding of the differences in the viral replication between the rabies virus rHEP-Flury and HEP-dG by observation of its spots in vivo. As with the result obtained by immunofluorescence staining of infected cells, the virus HEP-dG formed a greater number of brighter and more compact foci than the rabies rHEP-Flury at 12, 24 and 36 hpi (Fig. 6).

### Apoptosis

**Figure 7:**
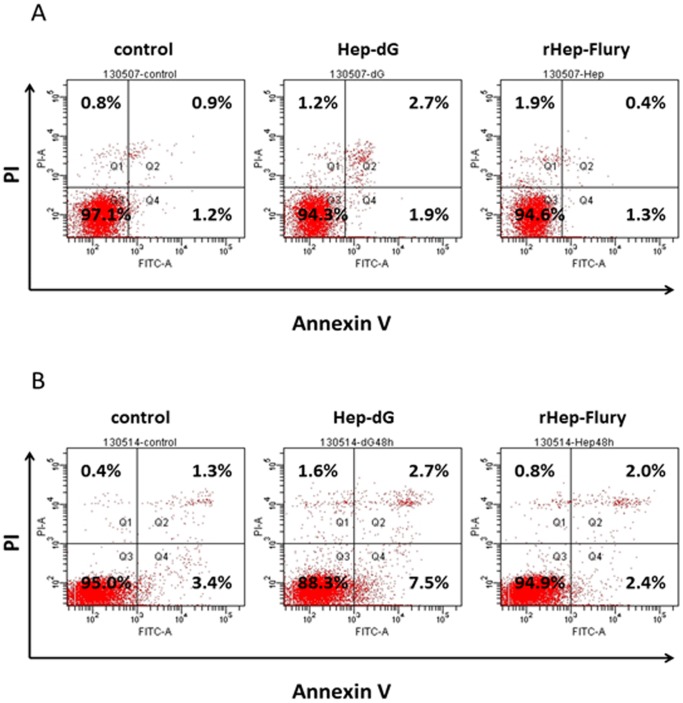
Apoptosis induced by virus HEP-dG and rHEP-Flury. The BHK cells and SK cells were infected with virus HEP-dG and rHEP-Flury. The apoptosis was analysed by Flow cytometry. The lower right part (Annexin V^+^/PI^−^) was considered as early stage of apoptotic cells and top right part (Annexin V^+^/PI^+^) was considered as late stage of apoptotic cells. The lower left part(Annexin V^−^/PI^−^) was considered as viable cells and the upper left part (Annexin V^−^/PI^+^) was considered as necrotic cells. BHK cells infected with virus HEP-dG and rHEP-Flury(A). SK cells infected with virus HEP-dG and rHEP-Flury(B). On the BHK and SK cells, HEP-dG induced more apoptosis than rHEP-Flury (*p*<0.01).

To analyze cell injuries induced by the virus HEP-dG strain, the BHK cells and SK cells infected with rHEP-Flury and Hep-dG were treated with Annexin V/PI. It was found that the virus HEP-dG induced 4.6% BHK cells apoptosis; however, rHEP-Flury only induced 1.7% BHK cells apoptosis (Fig. 7A). In addition, virus HEP-dG induced 10.2% SK cells apoptosis, and virus rHEP-Flury created 4.4% SK cells apoptosis (Fig. 7B). From this, HEP-dG induced more apoptosis than rHEP-Flury (*p*<0.01, Chi-square test using the Statistical Package for Social Sciences (SPSS®), version 17.0.) on the BHK and SK cells.

### Pathogenicity of recombinant virus HEP-dG strain in mice

**Figure 8:**
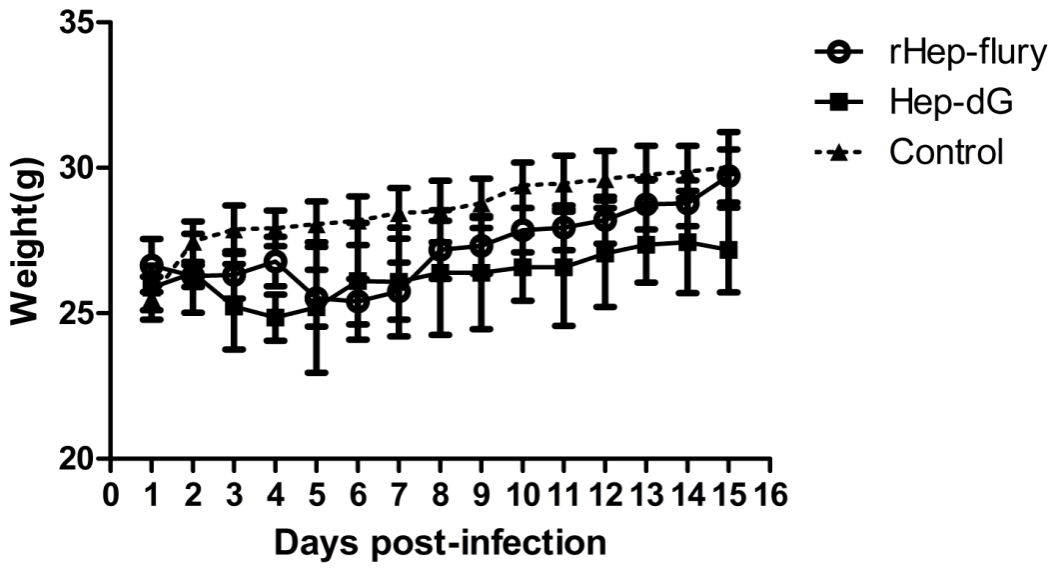
Body weigh changes in adult mice inoculated with the HEP-dG or rHEP-Flury. Ten mice per group were inoculated intracerebrally with HEP-dG (▪) or rHEP-Flury strain (**○**) at 5×10^4^ FFU per mouse or were mock-inoculated (▴). The changes are shown as ratios to body weights of mice at day 0 and taken as 1.

Mice at various ages were intracranially inoculated with HEP-dG or the parent virus. As summarized in Table 1, HEP-dG and rHEP-Flury were pathogenic only to young mice, causing the death in 0–4 week-old mice; but not in 6-week-old adult mice. In adult mice, the body weight was reduced slightly after infection (Fig. 8). The LD_50_ of HEP-dG (10^−2.60^/0.02 ml) was significantly different (*p*<0.001) from that of the rHEP-Flury (10^−5.61^/0.02 ml) in 3-days-old ddY mice (Table 2). These data suggested that HEP-dG encoding the double G-genes was less pathogenic than the parental rHEP-Flury. The statistical significance was analyzed by Chi-square test using the Statistical Package for Social Sciences (SPSS®), version 17.0.

**Table 1:** Pathogenicity of rabies virus in mice with different ages.

	Death		Survivorship	
Age(week-old)	HEP-dG	rHEP-Flury	HEP-dG	rHEP-Flury
0-1	10	10	0	0
1-2	10	10	0	0
2-3	9	10	1	0
3-4	8	9	2	1
6	0	0	10	10

**Table 2:** The LD_50_ of HEP-dG and rHEP-Flury in suckling mice.

	HEP-dG		rHEP-Flury	
Titer of virus	Mice	Death	Mice	Death
10^−1^	8	8	8	8
10^−2^	8	7	8	8
10^−3^	8	3	8	8
10^−4^	8	0	8	8
10^−5^	8	0	8	6
10^−6^	8	0	8	3
10^−7^	8	0	8	0

The LD_50_ of HEP-dG (10^−2.60^/0.02 ml) was significantly different (*p*<0.001) from that of the rHEP-Flury (10^−5.61^/0.02 ml).

### Immunogenicity of dG recombinant virus and protection against challenge in mice

**Figure 9:**
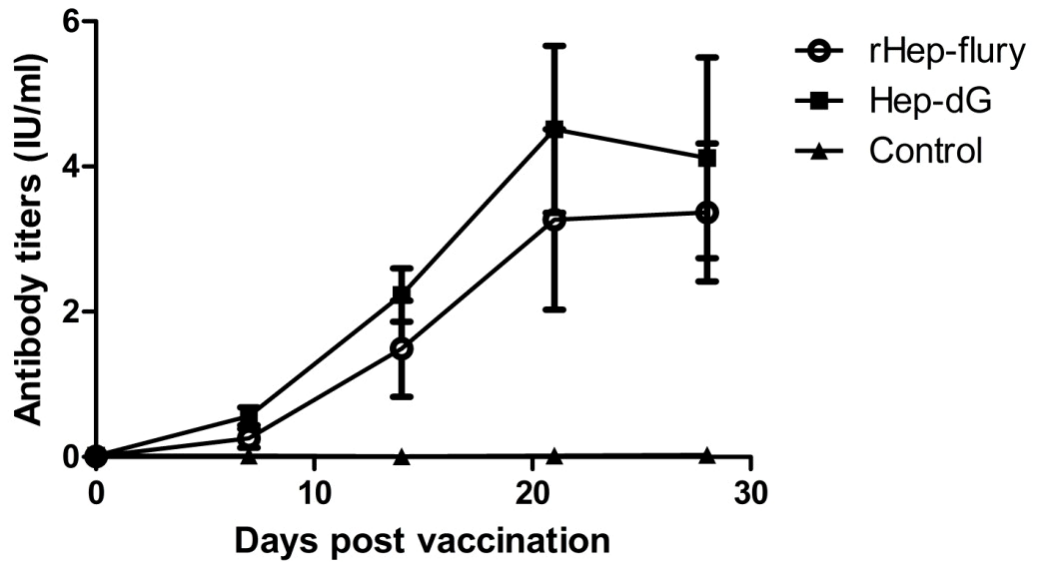
Antibody titers in mice immunized with inactivated RABV. Adult mice were immunized intramuscularly with inactivated vaccines (10^6^FFU/ml) of HEP-dG (▪) or rHEP-Flury (**○**) and bled at days 7,14,21,and 28. From the day 7, significantly higher VNA titers were induced by HEP-dG than by HEP-Flury strain (*p*<0.01).

To determine the immunogenicity, mice were immunized intramuscularly in the hind legs with 0.5 ml (10^6^FFU/ml) of HEP-dG or rHEP-Flury which was inactivated with 0.025% β-propiolactone. Blood were collected through the tail vein at weeks 1, 2, 3, and 4 after immunization and VNA determined. As summarized in Figure 9, both HEP-dG and rHEP-Flury induced high levels of VNA, reaching a peak at day 21 after immunization. Yet, HEP-dG induced higher levels of VNA (4.51 IU/ml) than the parental rHEP-Flury (3.27 IU/ml). From the day 7, significantly higher VNA titers were induced by HEP-dG than by HEP-Flury strain (*p*<0.01, independent-samples T Test using the Statistical Package for Social Sciences (SPSS®), version 17.0.).

**Figure 10:**
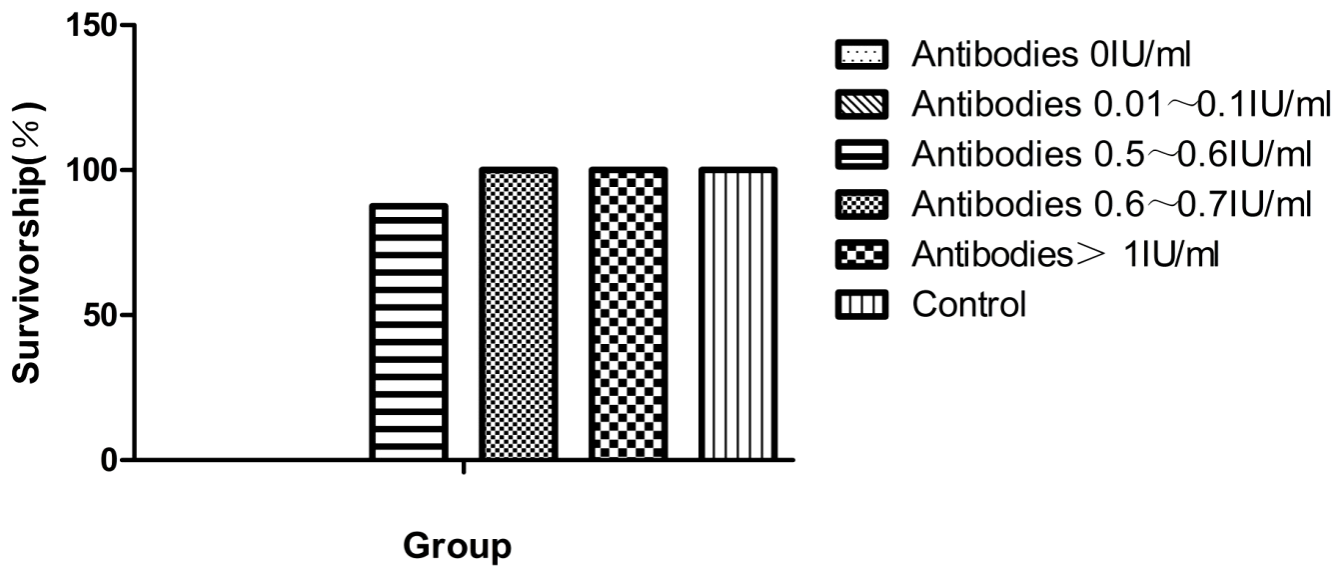
Protective immunity in mice. Mice were immunized with inactivated virus HEP-dG and blood was collected from the tail vein for determination of VNA. Mice were grouped according to VNA titers (0 IU/ml, 0.01–0.1 IU/ml, 0.5–0.6 IU/ml, 0.6–0.7 IU/ml and >1 IU/ml) and then challenged with a lethal dose of PV strain.

To determine if the VNA was protective in mice, five groups of mice whose antibody levels were 0 IU/ml, 0.01∼0.1 IU/ml, 0.5∼0.6 IU/ml, 0.6∼0.7 IU/ml, >1 IU/ml were selected for challenge with intracranial injection with 30 µl diluted RABV PV strain. Challenged mice were observed daily for four weeks. As summarized in Figure 10, 100% of the mice with VNA greater than 0.6 IU/ml were protected against challenge infection while 87.5% of the mice survived challenge when VNA was between 0.5 and 0.6 IU/ml. All mice succumbed to challenge when the VNA was lower than 0.5 IU/ml.

**Figure 11:**
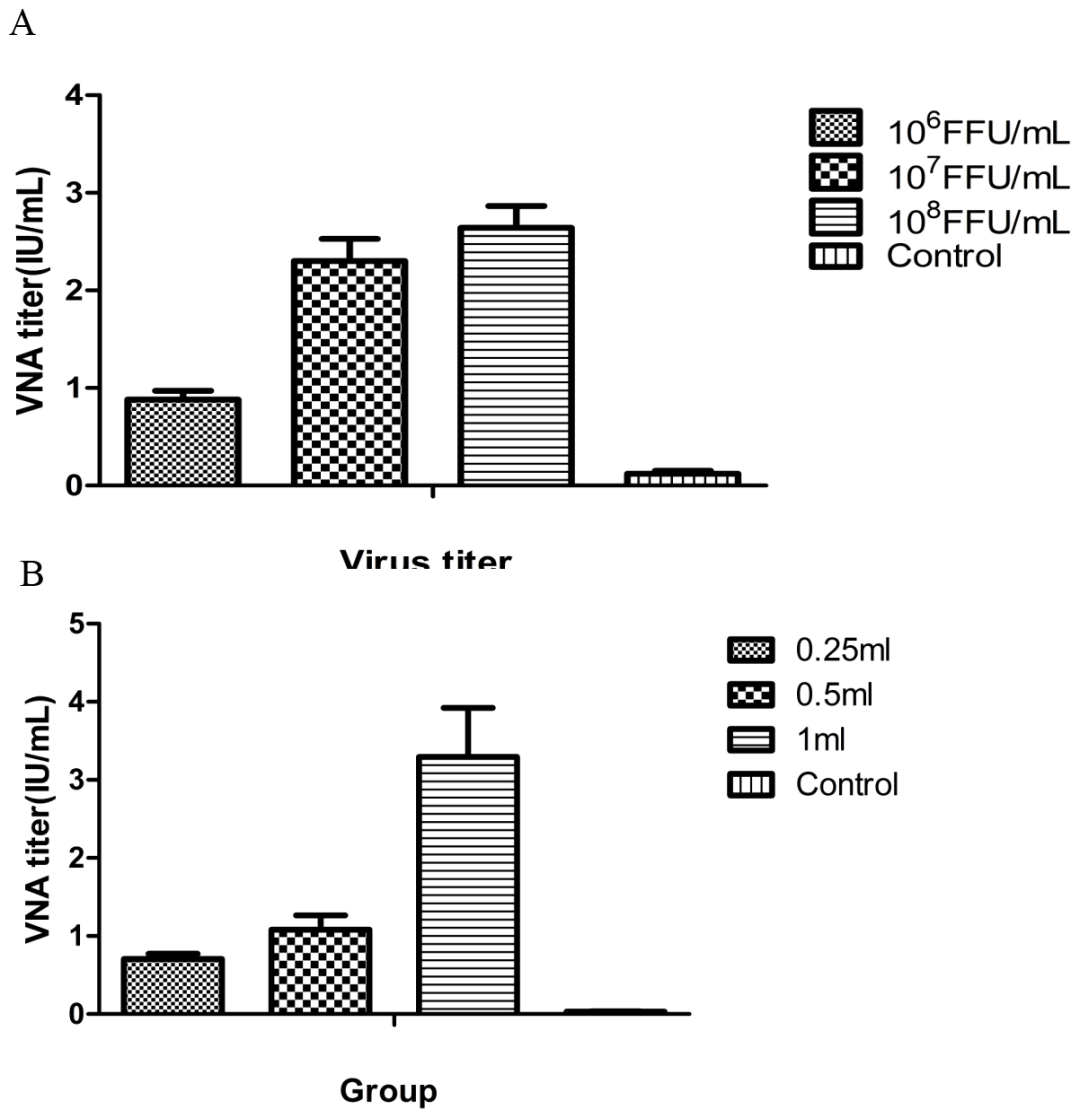
Relationship between glycoprotein and immunogenicity. Mice(A) and dogs(B) were immunized with vaccine containing different amounts of inactivated HEP-dG virus. At day 14 post-immunization blood were collected and VNA were determined.

To determine the relationship between G protein and immunogenicity. Mice were immunized with the vaccine containing 10^6^FFU/mL, 10^7^FFU/mL and 10^8^FFU/mL inactivated HEP-dG viruses. Blood were collected at day 14 after immunization and the antibody was detected. As shown in Figure 11(A) VNA improved when inactivated HEP-dG viruses increased. VNA titer induced by 10^8^FFU/mL HEP-dG virus was 2.64 IU/mL, about three times higher than those by with10^6^FFU/mL HEP-dG(*p*<0.05, independent-samples T Test using the Statistical Package for Social Sciences (SPSS®), version 17.0.).

### Immunogenicity of dG recombinant virus and challenge in dogs

To understand if the amount of G protein in vaccine exhibited positive correlation with VNA titer. Group of fifteen dogs were immunized respectively with 0.25 ml, 0.5 ml and 1.0 ml vaccine respectively containing 0.25×10^8^FFU/mL, 0.5×10^8^FFU/mL and 1.0×10^8^FFU/mL inactivated HEP-dG viruses. Serum were collected at day 14 after immunization and the antibody was detected. As shown in Figure 11(B), VNA titer also increased when inactivated HEP-dG viruses rised. VNA titer induced by 1.0 mL vaccine were 3.28 IU/mL, about three times higher than those by 0.25 mLvaccine(*p*<0.05, independent-samples T Test using the Statistical Package for Social Sciences (SPSS®), version 17.0.).

**Figure 12:**
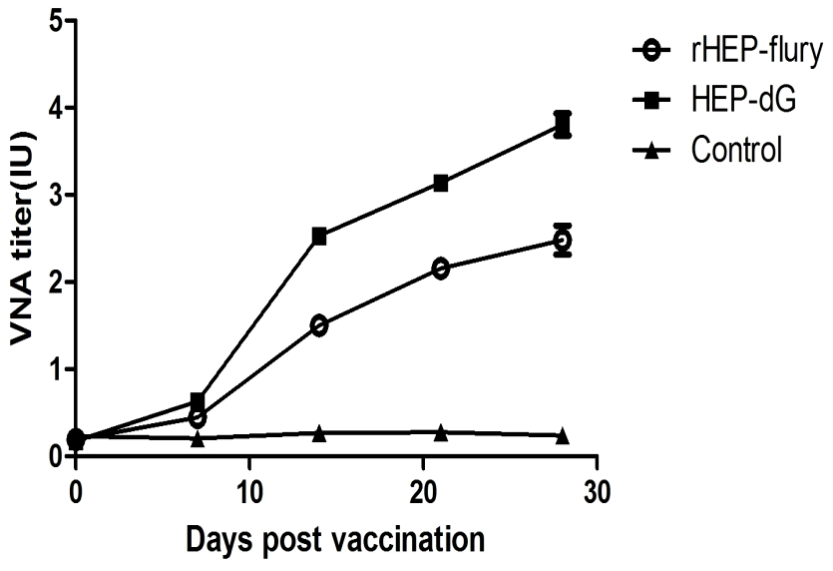
Kinetics of rabies antibodies after vaccination of dogs. Dogs were immunized with 1-dG (▪) or rHEP-Flury (**○**) at 1 ml per dog or mock-inoculated (▴). Blood were collected from jugular vein at days 7, 14, 21, and 28 after immunization and rabies VNA titers were determined.

To determine if the dG recombinant virus is immunogenic and can induce immune protection in dogs, three-month-old healthy, previously un-vaccinated dogs were immunized with 1 ml of inactivated viruses and challenged with 1 ml of RABV BD06 strain. Blood were collected from vein at days 7, 14, 21, and 28 after immunization and VNA determined. VNA titer gradually increased after immunization. At days 14, 21 and 28, VNA titers in dogs immunized with HEP-dG HEP-dG and rHEP-Flury were extremely significant different(*p*<0.001, independent-samples T Test using the Statistical Package for Social Sciences (SPSS®), version 17.0.) (Fig. 12).

**Figure 13:**
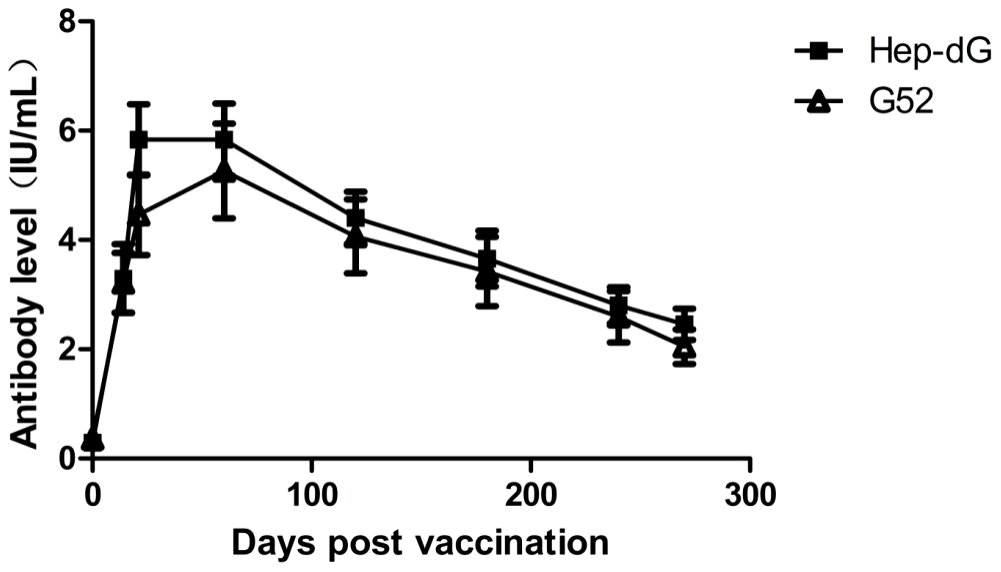
Comparison of antibody levels. Adult dogs were inoculated subcutaneously respectively with 1-dG (▪) or a commercial vaccine A(**Δ**). After immunization, each dog in groups A and B was bled intravenously at days 14, 21, 60, 120, 180, 240, 270 and the serum was used to determine VNA titers.

To compare the difference of antibody levels in dogs induced with HEP-dG vaccine and a commercial RV vaccine, sixteen adult dogs were inoculated with HEP-dG and a commercial vaccine, and bled at days 14, 21, 60, 120, 180, 240, 270. The results indicated that HEP-dG vaccine induced higher antibody titers in the dogs than the commercial vaccine on 14 and 21 day and the antibody titer levels were almost the same in dogs immunized with the two vaccines during the other months. (Fig. 13).

By day 90 after challenge, 33 out of 40 immunized dogs while only one of the 25 sham-immunized dogs survived the challenge. The protective rate was 82.5% in the immunized group (Table 3). Survival rates between immunized dogs and sham-immunized dogs were extremely significant different (p<0.001, Chi-square test using the Statistical Package for Social Sciences (SPSS®), version 17.0.).

**Table 3:** The result of protective immunity in dogs.

Group	Dog(number)	Death(number)	% survivorship
Group D	40	7	82.5
Group E	25	24	4

Three-month-old dogs of group C were inoculated with inactivated HEP-dG. At days 21 after immunization, the dogs in group D and E were challenged with 10^5^ lethal dose of BD06 strain. The survivorship of dogs vaccine with HEP-dG was significantly different (*p*<0.01) from control.

### Persistence of VNA in dogs

**Figure 14:**
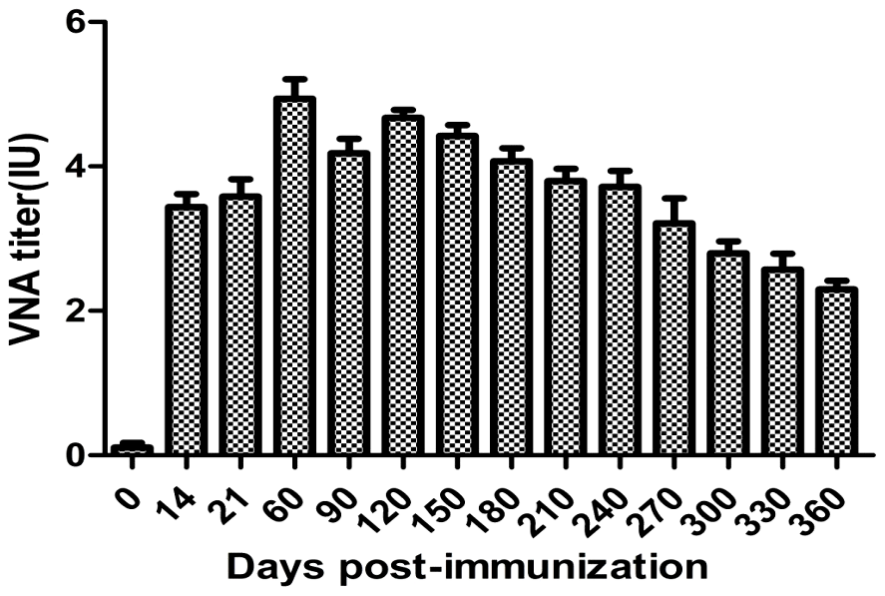
Persistence of VNA in dogs. Dogs were immunized with 1-immunization and rabies VNA titers determined.

To determine the persistence of VNA (above 0.6 IU/ml), 5 dogs were inoculated with the inactivated HEP-dG and observed for one year. Serum samples were collected at different time points after vaccination for VNA determination. The mean antibody titer was 3.438 at day 14 and reached peak at day 60 post-immunization. Then the VNA titer decreased slowly but still at 2.296 IU at day 360 post immunization. (Fig. 14)

## Discussion

One of the major ways to control human rabies is to control rabies in animals, especially in companion and wildlife animals [32]. Although live-attenuated rabies virus have been used to vaccinate domestic animals in the past, WHO recommends the use of inactivated rabies virus vaccines for domestic pets because live-attenuated rabies vaccine can occasionally revert to a virulent phenotype and cause rabies [11] [16] [17]. Rabies is still prevalent in China and causes more than 3,000 human deaths each year. This is majorly due to the fact that routine vaccination of domestic animals has not been undertaken, particularly in rural areas [9]. Although inactivated rabies virus vaccines have been imported to China for companion animal vaccination, their use has been limited due to the high cost of the vaccines. Currently, domestically produced inactivated rabies vaccines have not been licensed in China for animal vaccination and the only licensed rabies vaccines produced domestically are manufactured from the live-attenuated ERA strain [9]. It is imperative to develop inactivated rabies vaccines for routine animal vaccination in China that are affordable, safe and efficacious. In this present study, we report the development of a recombinant RABV vaccine by expressing two copies of the RABV G gene in the backbone of HEP-Flury strain. We have demonstrated that this recombinant virus grows to a high titer in BHK cells and immunization with inactivated preparation induces high VNA and protects mice and dogs from challenging infections.

The recombinant HEP-dG was constructed by reverse genetics technology that has been used to generate recombinant rabies viruses such as SAD-B19 [33], RC-HL [34], HEP-Flury [29], ERA [35], and wildlife isolate SHBRV-18 [36], SPBNGAS [37]. Expression of multiple copies of the G-gene has been reported previously for RC-HL [26], SAD B19 [22] [38], SPBNGAS [39]and Hep-Flury [23]. The rationale to express multiple copies of the G-gene is two fold. Firstly, the level of G-gene expression is inversely correlated with RABV pathogenicity due to its ability to induce apoptosis and innate immunity [40] [41] [42] [43] [44] [45]. Furthermore, the RABV G is the only surface protein of the RABV virion that is capable of inducing VNA and providing protective immunity [25]. Increased expression levels of the G can reduce RABV pathogenicity and simultaneously enhance immunogenicity. As has been shown previously for other RABV strains [22] [26], expression of two copies of the G-gene in HEP-Flury decreased RABV pathogenicity. The LD_50_ of recombinant virus HEP-dG was about 1000 times higher than that of parental virus. This observation is in agreement with previous studies, showing that the level of RABV G expression inversely correlated with pathogenicity [22] [26]. The mechanism is unknown. We speculate that the reason for this phenomenon is that an increase in the size of the genome reduces the amount of L gene because expression of genes is obligatorily sequential from a single 3′ promoter [46] [47]. Immunization of mice and dogs with inactivated dG induced higher VNA titers than the inactivated parental rHEP-Flury and developed protection against a virulent RABV strain.

A VNA titer equal to or above 0.5 IU has been used as an indicator for immunization and therefore adequate immunity for protection [48]. Correlation between this level of VNA and protection has been shown in mice and dogs [49]. To confirm the quality of VNA after immunization with HEP-dG, mice immunized with dG recombinant virus were challenged with a lethal dose of PV strain. It was shown that protection against challenge is dependent on the VNA level at the time of challenge. All the mice with VNA level equal to or more than 0.6IU/ml survived the challenge. These data suggest that HEP-dG is capable of inducing strong protective immunity. Furthermore, immunization of dogs with inactivated HEP-dG induced the production of high level of VNA and protection against a street RABV strain.

Another major finding in the present study is that the viral titer of recombinant virus HEP-dG in cultured cells is 100-fold higher than the parental virus rHEP-Flury after 6–8 passages. This was unexpected since higher level of the G expression led to apoptosis [22], which might reduce the virus yield. It is likely that more number of glycoprotein expression lead to receptor-mediated uptake more efficient and the virus replication in a larger number of cells, and thus this is definitely an advantage for vaccine production.

Inactivated RABV vaccines are usually mixed with adjuvant that can induce high antibody titers. In the present study, inactivated HEP-dG was mixed with 10%V/V Montanide PET GEL. It was found that such combination not only induced high level of VNA, but also resulted in persistence of VNA more than one year on dogs. Wide use of such vaccines can greatly reduce the numbers of vaccination, thus the cost associated with the vaccine and the labors. In summary, the recombinant virus HEP-dG strain can grow to higher titers when cultured in BHK-cells and is less pathogenic, but more immunogenic than the parent virus. Inactivation of the dG resulted in high VNA production and protection in mice and dogs. These data together indicate that the dG virus could be developed as a safe and efficacious inactivated virus vaccine in China.
